# Hodgkin lymphoma patients have an increased incidence of idiopathic acquired aplastic anemia

**DOI:** 10.1371/journal.pone.0215021

**Published:** 2019-04-05

**Authors:** Taylor Linaburg, Adam R. Davis, Noelle V. Frey, Muhammad R. Khawaja, Daniel J. Landsburg, Stephen J. Schuster, Jakub Svoboda, Yimei Li, Yuliya Borovskiy, Timothy S. Olson, Adam Bagg, Elizabeth O. Hexner, Daria V. Babushok

**Affiliations:** 1 Perelman School of Medicine, University of Pennsylvania, Philadelphia, PA, United States of America; 2 Department of Pathology and Laboratory Medicine, Hospital of the University of Pennsylvania, Philadelphia, Philadelphia, PA, United States of America; 3 Division of Hematology-Oncology, Department of Medicine, Hospital of the University of Pennsylvania, Philadelphia, PA, United States of America; 4 Division of Hematology-Oncology, Milton S. Hershey Medical Center, Penn State Cancer Institute, Hershey, PA, United States of America; 5 Department of Biostatistics and Epidemiology, Perelman School of Medicine at the University of Pennsylvania, Philadelphia, PA, United States of America; 6 Penn Medicine Corporate Information Services, University of Pennsylvania Health System, Philadelphia, PA, United States of America; 7 Division of Oncology, Department of Pediatrics, Children’s Hospital of Philadelphia, Philadelphia, PA, United States of America; European Institute of Oncology, ITALY

## Abstract

Idiopathic acquired aplastic anemia (AA) is a rare lymphocyte-mediated bone marrow aplasia. No specific mechanisms or triggers of AA have been identified. We recently observed several patients who developed AA after Hodgkin lymphoma (HL). We hypothesized that the co-occurrence of HL and AA is not random and may be etiologically significant. To test this hypothesis, we determined the incidence of AA in HL patients at our institution. We identified four patients with co-occurring HL and AA, with the incidence of AA in HL patients >20-fold higher compared to the general population. We identified 12 additional patients with AA and HL through a systematic literature review. Of the 16 total patients,15 (93.8%) developed AA after or concurrent with a HL diagnosis. None of the patients had marrow involvement by HL. Five of 15 patients were in complete remission from HL at the time of AA diagnosis, and six had a concurrent presentation with no prior cytotoxic therapy, with diagnostic timeframe information unavailable for four patients. The median interval between HL diagnosis and AA onset was 16 months, ranging from concurrent to 14 years after a HL diagnosis. The median survival after AA diagnosis was 14 months (range: 1 month to 20 years). Our results show that patients with HL have a higher incidence of AA compared to the general population and suggest that HL-related immune dysregulation may be a risk factor for AA. Better recognition and management of AA in HL patients is needed to improve outcomes in this population.

## Introduction

Idiopathic acquired aplastic anemia (AA) is a rare, life-threatening disease characterized by pancytopenia and a hypocellular marrow that is caused by lymphocyte-mediated destruction of hematopoietic stem and progenitor cells [1]. Treatment of AA relies on lymphocyte-directed immunosuppression, administered either as anti-thymocyte globulin (ATG) and cyclosporine A (CsA) or as part of a bone marrow transplant (BMT) conditioning regimen [2]. Despite recent progress in outcomes of AA patients, the understanding of the autoimmune mechanism in AA remains limited; no triggers of autoimmunity in AA have been identified, and there are no known prevention strategies for this condition. The diagnosis of AA is established clinically through the systematic exclusion of mimicking entities, with T-cell immunosuppressive treatments continuing to be largely empiric.

Several clinical observations have established a link between AA and other conditions that suggest a shared pathogenesis, most notably seronegative hepatitis [3] which precedes AA in ~10–15% of patients, and a less frequent association with eosinophilic faciitis [4, 5]. Epidemiologic and genetic association studies have identified several factors associated with a modest increase in the risk of AA, such as exposures to benzene, pesticides and waterfowl [6, 7], and genetic polymorphisms in cytokine genes, metabolic enzymes, and human leukocyte antigen (HLA) alleles [8–15]. More recently, a link between AA and lymphoid neoplasms, including T-cell large granular lymphocyte leukemia [15] and B-cell leukemias and lymphomas [16, 17] has also been proposed. However, there are no studies of the incidence of AA in B-cell or T-cell neoplasms and no specific connection to B-cell neoplasms has been established.

Interestingly, we recently observed several patients who developed AA following a diagnosis of HL, a distinctive B-cell neoplasm associated with an abnormal immune response [18]. Because AA and HL are both very rare—the incidence of AA in Western countries is 2.2 cases per million per year and up to 7 cases per million per year in Asia [6, 19], while the incidence of HL is ~2.6 cases per 100,000 per year [20]—we hypothesized that their co-occurrence is etiologically linked and that HL patients are more likely to develop AA compared to the general population.

To test for an association between AA and HL, we determined the incidence of AA in patients with HL seen at our institution between 2005 and 2018 and compared it to the incidence of AA in the general population. Strikingly, patients with HL had at least a 20-fold higher incidence of AA than the general population. To comprehensively evaluate the characteristics of AA in patients with HL, we expanded our institutional cohort by including published cases identified through a systematic literature review. In the following analysis, we present the clinical and pathologic characteristics of AA when presenting in patients with HL, as well as treatment strategies and patient outcomes seen with this rare and difficult-to-treat population.

## Methods

### Patients and study oversight

Data collection and analysis were performed with the approval of the Institutional Review Board of the University of Pennsylvania. Cases of co-occurring HL and AA were identified by the search of electronic medical records of patients treated at the Hospital of the University of Pennsylvania between 2005 and 2018 using ICD-9 and ICD-10 diagnosis codes (Tables A-C in S1 Appendix). 2801 patients with a diagnosis of HL and 6842 patients with a diagnosis of AA or pancytopenia were identified, with 125 patients carrying both AA or pancytopenia and HL diagnoses. This 125 patient cohort was then used to identify patients who met the full diagnostic criteria for both HL and AA through the comprehensive manual review of medical charts. After completion of the diagnostic review, bone marrow histology was assessed in a blinded manner by a hematopathologist for patients who were entered into the study. The diagnosis of HL was established according to the 2016 World Health Organization (WHO) classification [21, 22]. The diagnosis of AA was established according to the International Study of Agranulocytosis and Aplastic Anemia criteria and required systematic exclusion of mimicking entities [2, 19, 23]. After the manual record review, 91 of the 125 patients were confirmed to have a diagnosis of HL (72.8% true positive rate of HL based on ICD-9 and ICD-10 code search for HL) and 4 of 125 patients were confirmed to have a diagnosis of AA (3.2% true positive rate of AA based on ICD-9 and ICD-10 code search for AA or pancytopenia).

### Case presentations

**Fig 1:**
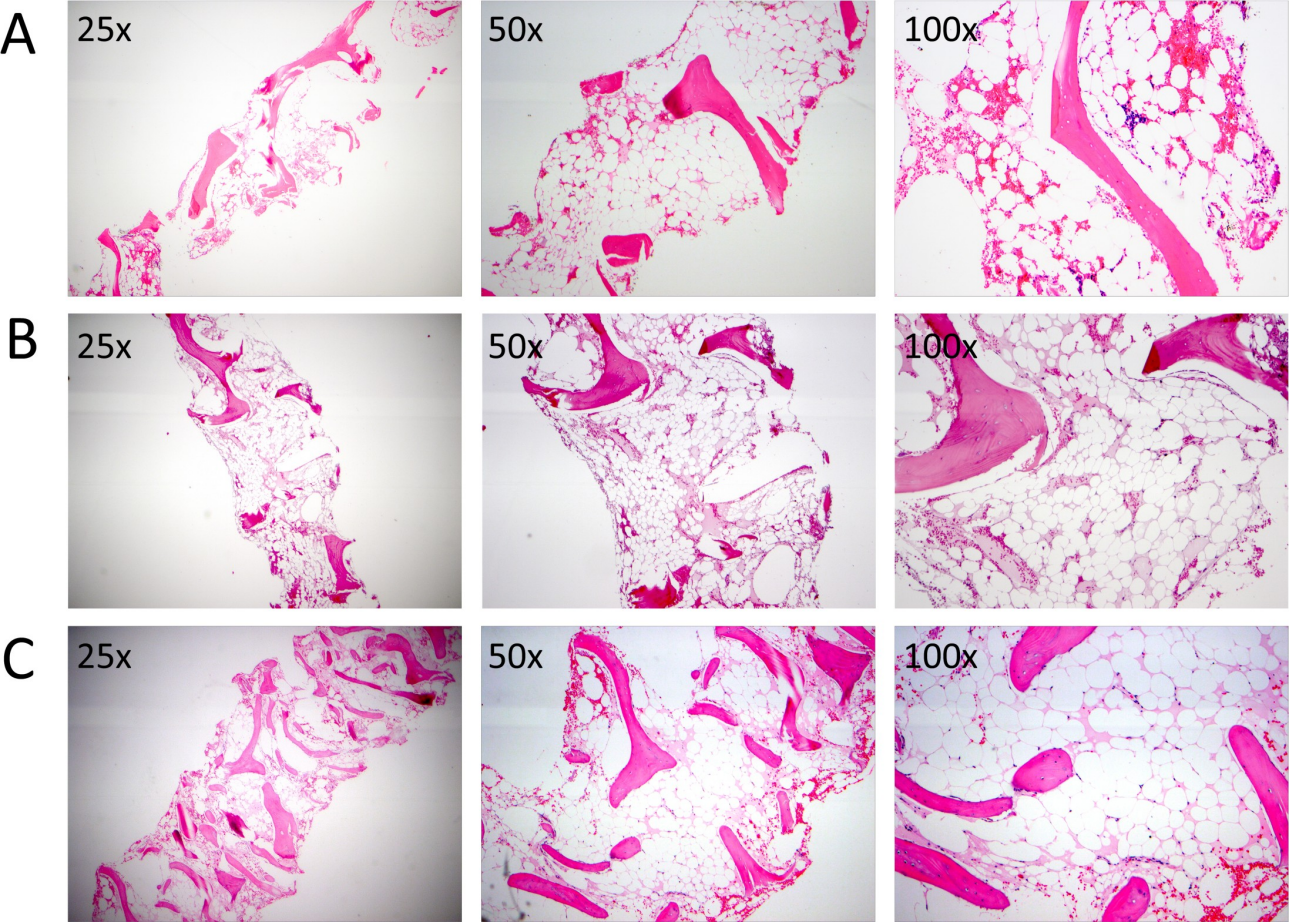
Bone marrow histology at the time of aplastic anemia diagnosis in patients with previous history of Hodgkin Lymphoma. The hematoxylin and eosin (H&E) stained bone marrow histology sections at 25x, 50x, and 100x magnification reveal severely reduced cellularity and reduced trilineage hematopoiesis of Patient 1 (A), Patient 2 (B), and Patient 3 (C).

Patient 1 is a South Asian Indian male who, at the age of 34 years, was diagnosed with HL and achieved a complete remission after receiving ABVD (doxorubicin, bleomycin, vinblastine, dacarbazine) followed by ASHAP (doxorubicin, methylprednisone, high dose cytarabine, cisplatin). Fourteen years after the diagnosis of HL, the patient developed severe pancytopenia and was found to have a markedly hypocellular bone marrow (Table 1, Fig 1A). There was no evidence of HL recurrence. Cytogenetics were normal, and there was no dysplasia. Work-up did not reveal any infectious or nutritional causes of pancytopenia. Epstein-Barr Virus (EBV) serologies were consistent with a prior but not current infection. Paroxysmal Nocturnal Hemoglobinuria (PNH) flow cytometry was negative. Past medical history was notable for testicular carcinoma in the remote past treated by orchiectomy as well as etoposide and cisplatin-based chemotherapy, with complete blood count recovery after therapy. Based on AA diagnostic and severity criteria, a diagnosis of severe AA was made. The patient received immunosuppressive therapy with horse ATG (hATG) and cyclosporine A (CsA) with no response at six months, followed by the addition of eltrombopag with no response. Eleven months after the initial diagnosis, the patient received a 9/10-matched unrelated donor bone marrow transplant (MUD-BMT) for severe AA with Fludarabine (Flu)/Cyclophosphamide(Cy)/rabbit ATG (rATG) conditioning and total-body irradiation of 200 cGy. Graft versus host disease (GVHD) prophylaxis was methotrexate and tacrolimus. The patient’s post-transplant course was complicated by primary graft failure, with the patient ultimately dying from infectious complications after transplant.

**Table 1:** Clinical and pathologic characteristics of patients with AA and HL.

Patient #	HL subtype	Bone marrow histology at AA diagnosis	Cytogenetics	T-cell receptor rearrangement	NGS for myeloid malignancy-associated mutations	PNH flow cytometry	HLA alleles	Inherited marrow failure testing, if available
**Patient 1**	n/a	Markedly hypocellular (5% cellularity) with markedly decreased trilineage hematopoiesis.	46,XY[20] (diagnosis); evolved to 46,XY,der(21)t(3;21)(q11.2;p13)[12] after immunosuppression	Polyclonal	Negative	Negative	A*01:01, A*24:07, B*35:02, C*04:01, DR*11:04	n/a
**Patient 2**	nodular sclerosis	Markedly hypocellular marrow (<5% cellularity) with markedly decreased trilineage hematopoiesis.	46,XY [20]	Polyclonal	Negative	Negative	A* 68:02, A*02:01; B* 14:02, B*18:01, C*08:02, C*07:01, DR* 13:03, 08:01	Chromosome breakage: normal; Lymphocyte telomere length: very high
**Patient 3**	nodular sclerosis	Markedly hypocellular marrow (<5% cellularity) with markedly decreased trilineage hematopoiesis.	46,XY[1]; poor growth	n/a	n/a	Negative	A*2, 24; B*39, 51; Cw*7, 15, DR*4, 13, DQ*6, 8	n/a
**Patient 4**	nodular sclerosis	Hypocellular marrow (20%), with foci of erythropoiesis and myelopoiesis.	Normal^a^	n/a	n/a	n/a	A*1, 3; B*35, 57; Cw*02:02; Cw*06:02	n/a

HL, Hodgkin Lymphoma; AA, Idiopathic Acquired Aplastic Anemia; NGS, Next-Generation Sequencing; HLA, Human Leukocyte Antigen; PNH, Paroxysmal Nocturnal Hemoglobinuria.

^a^For patient 4, results of metaphase cytogenetics from the time of AA diagnosis are not available, however, karyotype after immunosuppression was normal (46,XY,inv(9)(p11q13)c[20]).

Patient 2 is a Caucasian male who, at the age of 28, was diagnosed with a gastric mucosa-associated lymphoid tissue (MALT) lymphoma and achieved a complete remission with four cycles of rituximab. Two years later, the patient developed progressive pancytopenia, supraclavicular and mediastinal adenopathy, and drenching night sweats. Initial lymph node biopsies revealed no malignancy. The patient’s bone marrow was hypocellular with normal cytogenetics and no lymphomatous involvement. The patient was initially treated with empiric steroids, intravenous immunoglobulin (IVIG) and eltrombopag without response. Subsequent positron emission tomography-computed tomography (PET-CT) demonstrated increasing Fluorodeoxyglucose (FDG)-avid lymphadenopathy in the mediastinum and supraclavicular areas, and a femoral lesion. An excisional biopsy of a cervical lymph node was diagnostic for HL, nodular sclerosis type. The patient developed progressive neutropenia and continued to be red cell and platelet-transfusion dependent, with a bone marrow biopsy showing a hypocellular bone marrow with 5% cellularity, no dysplasia and no evidence of involvement by HL (Fig 1B, Table 1). No infectious or nutritional causes of pancytopenia were identified. EBV polymerase chain reaction (PCR) was negative. PNH flow cytometry was negative. T-cell receptor gamma gene rearrangement showed no clonal T-cell process. Lymphocyte telomere length and chromosome breakage studies showed no evidence of a short telomere syndrome or Fanconi Anemia. Because of the concomitant HL and AA presentation, he was not able to receive standard therapy for either condition. After progressing on brentuximab vedotin, MUD-BMT was recommended. However, he died of neutropenic sepsis with fungal pneumonia 17 months after initial presentation, following one cycle of salvage ICE (ifosfamide, carboplatin, etoposide) chemotherapy in an attempt to control his HL prior to BMT.

Patient 3 is a South Asian Indian male who, at the age of 35 years, was diagnosed with stage IVB nodular sclerosis HL. He achieved a complete remission after receiving six cycles of ABVD. Ten years after his initial diagnosis of HL, he developed severe thrombocytopenia and was initially empirically treated with prednisone and IVIG without significant improvement. Subsequent bone marrow biopsy showed a markedly hypocellular marrow with essentially absent trilineage hematopoiesis and without evidence of malignancy (Fig 1C, Table 1). Work-up for infectious etiologies was unrevealing. A presumptive diagnosis of severe AA was made. The patient was treated with immunosuppressive therapy with hATG and CsA. Four months after his AA presentation, the patient developed a rising white count with immunophenotypically aberrant cells indicative of a therapy-related myeloid neoplasm (TRMN) in the setting of prior cytotoxic therapy. The patient died shortly afterwards from infectious complications.

Patient 4 is a Caucasian male diagnosed with severe AA at the age of 32 years who achieved remission after immunosuppressive therapy with ATG and CsA. Seven years after initial therapy, he developed falling blood counts and splenic enlargement. Splenectomy revealed splenic MALT lymphoma. Six years later, the patient developed adenopathy and night sweats. PET-CT showed extensive cervical lymphadenopathy and numerous foci of intense FDG activity within the skeleton; lymph node biopsy demonstrated HL, nodular sclerosis subtype. He was treated with six cycles of ABVD, achieving a complete remission and remains well without cytopenias 80 months after completion of chemotherapy.

### Calculation of incidence and prevalence of AA in HL patients and statistical analysis

The incidence of AA in HL patients was determined as the number of HL patients diagnosed with AA divided by the AA-free person-years of HL patients treated at the Hospital of the University of Pennsylvania between 2005 and 2018. The AA-free person-years in HL patients were conservatively estimated as follows. 2801 total HL patients (determined from HL ICD-9/-10 electronic medical record review) x 72.8% (true positive rate of HL determined by manual review of patients identified by ICD-9/-10 codes) resulted in ~2040 patients with HL. Of these patients, 2036 patients did not have a diagnosis of AA, and their disease-free person-years were calculated as the 13 years of the study period x 2036 patients. For the four patients who had HL and AA, the disease-free person-years were manually calculated based on the number of years within the study period prior to AA diagnosis (11, 12, 6, 0, for a total of 29). The prevalence of AA in HL patients was calculated as the number of AA diagnoses in HL patients divided by the total number of HL patients evaluated during the study period.

We found the estimated incidence of AA in HL patients to be ~151 cases per million HL patients per year (4 in 26,497 person-years, 95% confidence interval (CI) [41 per million, 385 per million]). We compared this estimated incidence to the published incidence of AA in the general population of 2.2 cases per million per year in Western countries to ~7 cases per million per year in Asia [6]^,^[19] [6, 19] using the two-tailed Exact test based on Poisson distribution using the Stata 15 software (StataCorp LLC, College Station, TX) with a significance level of p<0.05. We found the prevalence of AA in our institutional cohort to be 0.2% (4 of 2040 patients, 95% CI of [0.05%, 0.51%]), and compared this value to the prevalence of AA in patients with HL from published studies [24, 25] using the two-tailed Fisher’s exact test with a significance level of p<0.05.

### Systematic literature review

This systematic review was performed according to the Preferred Reporting Items for the Systematic Review and Meta-Analyses (PRISMA) Statement [26] (Figure A in S1 Appendix). The inclusion criteria for article selection were that the abstracts must mention a patient or a patient population with HL or Hodgkin disease (HD) as well as AA or pancytopenia. Full-text articles were then reviewed and excluded if the article included: a) patients with AA with no evidence of a HL or HD diagnosis, b) patients with HL or HD with no evidence of an AA or unexplained pancytopenia diagnosis (e.g., we excluded HL complications where an alternative etiology of pancytopenia was likely, such as post-chemotherapy myelosuppression, infections, myelodysplastic syndrome, acute leukemia, and hemophagocytic syndrome), c) relevant cases already described in previously identified research articles. This search strategy was used to identify appropriate abstracts and subsequent articles from Pubmed and was supplemented by a manual search of Google Scholar. Candidate abstracts were identified using keywords “Hodgkin lymphoma,” “Hodgkin disease,” “Aplastic anemia,” and “pancytopenia,” excluding the term “Non-Hodgkin.” No date or language limits were applied to the database search. Non-English language articles were translated and reviewed. Two authors (T.L. and D.B.) performed the search and evaluated abstracts independently.

### Analysis of clinical characteristics of AA in patients with HL

To the extent available within published reports and medical records, we collected the following variables for all patients with diagnoses of AA and HL: patient’s sex, age at HL and AA diagnosis respectively, interval between HL and AA diagnoses and order of diagnoses, HL subtype and Ann Arbor stage, HL treatment prior to AA diagnosis, HL outcome, HL status at AA diagnosis, severity of AA, treatment offered for AA, AA outcome, other pertinent neoplasms, cytogenetics, family history, duration of follow-up and overall survival.

## Results

### Patients with HL have an increased incidence of AA compared to the general population

**Fig 2:**
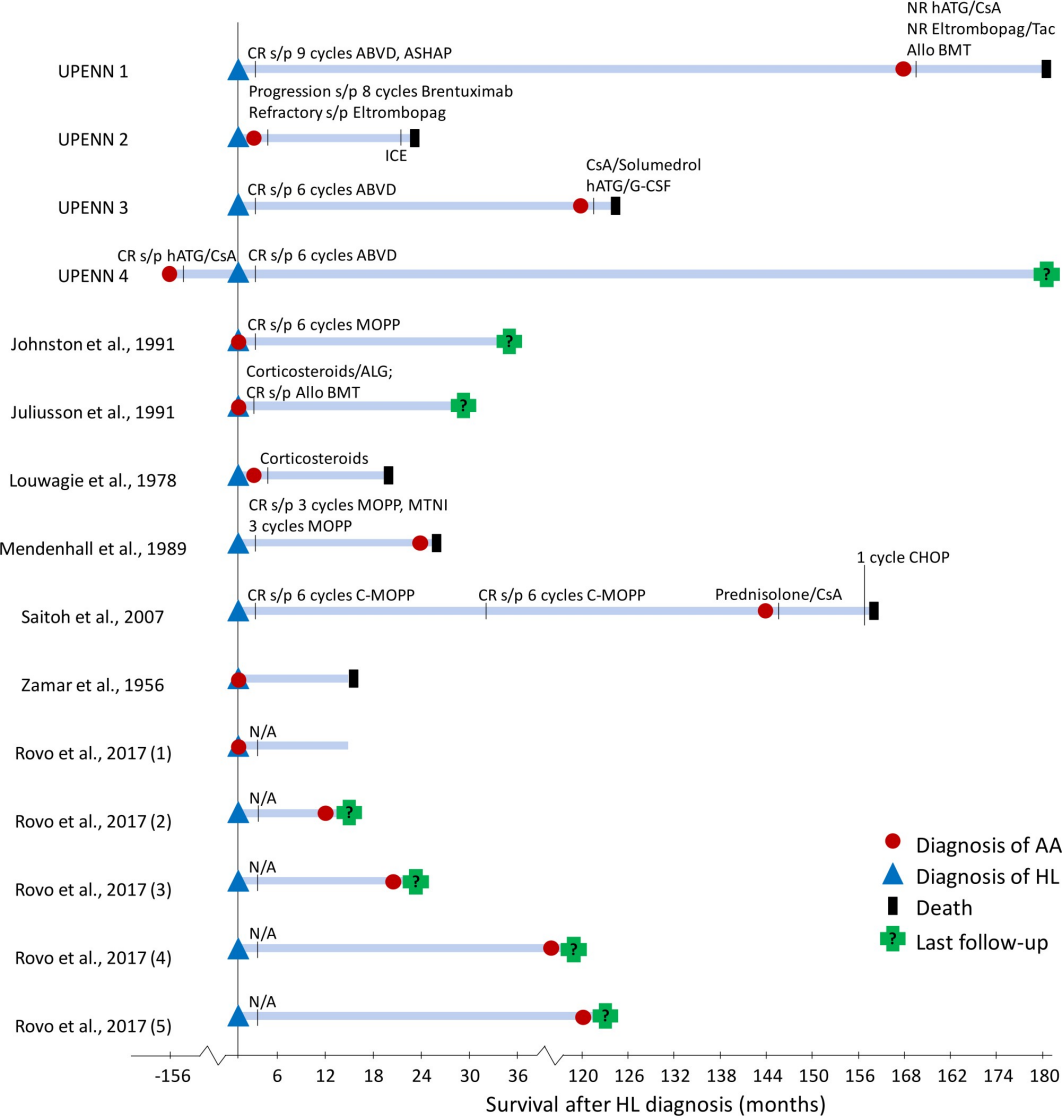
Clinical Course of patients with Hodgkin Lymphoma and Aplastic Anemia. A schematic diagram demonstrating the clinical course of 15 patients with diagnoses of HL and AA. Patient reported by Brusamolino et al. is not shown because of incomplete clinical information. Blue triangle, HL diagnosis; red circle, AA diagnosis; black triangle, death; green cross, last follow-up. Treatments for HL and AA are denoted by black vertical lines, with treatment and response listed above the respective line. HL, Hodgkin Lymphoma; AA, Idiopathic Acquired Aplastic Anemia; ABVD, Doxorubicin, Bleomycin, Vinblastine, Dacarbazine; ASHAP, Doxorubicin, Solumedrol, Cytarabine, Cisplatin; MOPP, Mechlorethamine, Vincristine, Procarbazine, Prednisone; MTNI, Modified Total Nodal Irradiation; C-MOPP, Cyclophosphamide, Vincristine, Procarbazine, Prednisone; CHOP, Cyclophosphamide, Doxorubicin, Vincristine, Prednisone; Allo BMT, Allogenic Bone Marrow Transplant; hATG, Horse Antithymocyte Globulin; ATG, Antithymocyte Globulin; ALG, Antilymphocyte Globulin, CsA, Cyclosporine A; Tac, Tacrolimus; G-CSF, Granulocyte Colony Stimulating Factor; CR, Complete Response; PR, Partial Response; NR, No Response.

Of the 2040 patients with a diagnosis of HL evaluated at the Hospital of the University of Pennsylvania between 2005 and 2018, four patients were also diagnosed with AA (Figs 1 and 2, Tables 1 and 2). All four patients were male, and three of the four presented with AA after or concurrent with a HL diagnosis. The median age at AA presentation was 39 years (range: 31 to 49 years). All patients had severe or very severe AA, and none had evidence of active EBV infection, myelodysplasia, or features consistent with an occult inherited bone marrow failure syndrome (see methods for detailed case descriptions).

**Table 2:** Clinical characteristics of patients with hodgkin lymphoma and aplastic anemia.

Patient	HL type Stage (if available)	HL Treatment pre-AA diagnosis	HL Status at AA diagnosis	Age at AA diagnosis/Sex	Interval between HL and AA diagnoses	AA treatment	Survival after AA diagnosis	Status of AA at last follow-up
**Patients reported in this study**								
**UPENN 1**	N/A; Stage II^a^	ABVD, ASHAP	CR	49/M	14 years	hATG/CsA; Eltrombopag/Tac; Allo BMT	12 months	Refractory; died of primary graft failure after Allo BMT
**UPENN 2**	nodular sclerosis;Stage IV	None	Progression s/p 8 cycles Brentuximab	31/M	≤ 2 months; concurrent	Eltrombopag	17 months	Refractory; died of AA complications
**UPENN 3**	nodular sclerosis;Stage IVB	ABVD	CR	46/M	10 years	CsA/Solumedrol; hATG/G-CSF	4 months	Refractory; transformed to TRMN
**UPENN 4**	nodular sclerosis; Stage IVB	None^b^	CR s/p 6 cycles ABVD	32/M	AA preceded HL by 13 years	CsA/ATG	Not reached (>20 years)	CR s/p CsA
**Patients from the Systematic Literature Review**								
**Brusamolino et al., 1994**	N/A	MOPP/ABVD	Presumed CR	N/A	N/A	Supportive	N/A	Died of hemorrhage
**Johnston et al., 1991**	lymphocyte depleted; Stage IIIB	MOPP	CR	31/F	0 months; concurrent	Supportive	Not reached (34 months as of publication)	Active AA
**Juliusson et al., 1991**	nodular sclerosis; Stage IIA	None	CR s/p Allo BMT	46/M	0 months; concurrent	Corticosteroids/ALG; Allo BMT	Not reached (16 months as of publication)	CR s/p Allo BMT
**Louwagie et al., 1978**	lymphocyte predominant; Stage I	None	Concurrent	24/M	≤ 2 months; concurrent	Corticosteroids	8 months	Died of cerebral hemorrhage
**Mendenhall et al., 1989**	N/A; Stage IIIB	MOPP, MTNI	CR	14/M	2 years	Supportive	1 months	Died within 1 month of AA diagnosis
**Rovo et al., 2017 (5 cases)**	1 nodular sclerosis; 2 lymphocyte predominant; 1 mixed cellularity; 1 unspecified	Unknown	Unknown	Unknown	Concurrent; 12 months; 20 months; 8 years; 10 years	Unknown	Unknown	Unknown
**Saitoh et al., 2007**	nodular sclerosis; Stage IVA	C-MOPP	CR	39/F	12 years	Prednisolone/CsA	14 months	Refractory; died of GI hemorrhage
**Zamar et al., 1956**	N/A	None	Concurrent	12/M	0 months; concurrent	Supportive	3 months	Died 3 months after AA diagnosis

N/A, Not Available; HL, Hodgkin Lymphoma; AA, Idiopathic Acquired Aplastic Anemia; ABVD, Doxorubicin, Bleomycin, Vinblastine, Dacarbazine; ASHAP, Doxorubicin, Solumedrol, Cytarabine, Cisplatin; MOPP, Mechlorethamine, Vincristine, Procarbazine, Prednisone; C-MOPP, Cyclophosphamide, Vincristine, Procarbazine, Prednisone; MTNI, Modified Total Nodal Irradiation; CR, Complete Remission; PR, Partial Response; Allo BMT, Allogenic Bone Marrow Transplant; ATG, Antithymocyte Globulin; hATG, Horse Antithymocyte Globulin; CsA, Cyclosporine A; Tac, Tacrolimus; G-CSF, Granulocyte Colony Stimulating Factor; ALG, Antilymphocyte Globulin; TRMN, Therapy-Related Myeloid Neoplasm; GI, Gastrointestinal.

^a^Incomplete staging information.

^b^AA diagnosed and treated before onset of HL.

Using a conservative assumption that all HL patients who were seen at the Hospital of the University of Pennsylvania between 2005 and 2018 had their AA diagnosis captured within the University of Pennsylvania medical record, the estimated incidence of AA in HL patients was ~151 cases per million HL patients per year (4 in 26,497 person-years, 95% CI [41 per million, 385 per million]). Compared to the published epidemiologic data for AA incidence in the general population of 2.2 cases per million per year in Western countries to ~7 cases per million per year in Asia [6]^,^[19], we estimate that patients with HL have at least a 20-fold higher incidence of AA compared to the general population (range: 21 to 68-fold higher; p = 0.0001). Because of the strikingly high incidence of AA in our HL patient cohort, we wondered whether the increased AA incidence could be explained by atypical referral patterns to our tertiary care center. To address this question, we compared the observed prevalence of AA in our institutional cohort to that reported in prospective studies of long-term outcomes in HL patients; a prevalence comparison was performed because the incidence data for AA in published HL cohorts was not available. We found that the prevalence of AA in our institutional cohort (0.2%, 4 of 2040, 95% CI [0.05%, 0.51%]) was not different from the prevalence of AA in prospective HL cohorts, where AA was reported at frequencies of 0.7% (1 of 138) [24] and 0.5% (1 of 200) [25] (p = 0.205). Because no published data on the prevalence of AA in B cell neoplasms are available, we compared the prevalence of AA in patients with HL to the AA prevalence in patients with chronic lymphocytic leukemia (CLL) also seen at our institution. The prevalence of AA in HL patients was higher than in patients with CLL (0.2% (4 of 2040) compared to 0.04% (1 of 2802)), although the small number of patients limited the strength of this comparison (p = 0.169).

### Twelve additional cases of AA in HL patients were identified through a systematic literature review

To more comprehensively evaluate the characteristics of AA when presenting in patients with HL, we performed a systematic literature review. Using a literature search strategy in accordance PRISMA best practices for systematic reviews and meta-analyses, we identified 172 unique abstracts of which 25 full-text articles fulfilled the eligibility criteria (Figure A in S1 Appendix). After a detailed review, we identified eight articles that included a total of 12 cases where HL patients were also diagnosed with AA (Fig 2, Table 2). In all cases, AA was diagnosed either concurrently with HL or when the patient was in remission from a previous HL diagnosis.

### Clinical and pathologic characteristics of AA occurring in HL patients

Of the 16 patients with AA and HL (four from our institution and 12 identified through the systematic literature review), the median age at AA diagnosis was 31.5 years (range: 12 to 49 years) with eight of the ten patients who had evaluable demographic information being male (80%). Fifteen of the 16 patients (93.8%) developed AA after or concurrent with a HL diagnosis (Table 2). From the 15 cases for which the interval between the two diagnoses was available, six patients (40.0%) developed AA concurrently with HL (i.e. identified simultaneously or recognized in short succession within two months of diagnosis of HL), three patients (20.0%) developed AA within two years of HL diagnosis, five patients (33.3%) developed AA over five years after HL diagnosis, and one patient (6.7%) had an AA diagnosis that preceded HL by 13 years. The median interval between HL diagnosis and AA onset was 16 months, ranging from concurrent presentation to a maximum of 14 years after HL.

Of the 11 patients with available HL pathologic subtype, six had nodular sclerosis (54.6%), one had lymphocyte depleted HL (9.1%), one had mixed cellularity HL (9.1%), and three patients presented with lymphocyte predominant HL (27.3%) (Table 2). HL subtype was not available for five patients. Six patients were diagnosed with advanced stage (stage III-IV), and three patients had early-stage HL; staging information was not available for seven patients (Table 2). None of the patients had HL involvement of their bone marrow at the time of AA diagnosis.

At the time of AA diagnosis, AA was graded as severe or very severe in all patients for whom severity grading information was available (eight of eight patients). Cytogenetic analysis at the time of AA diagnosis was normal in all evaluable patients (five of five patients). Among the four cases from our institution, two had remote diagnoses of MALT lymphoma (one treated by splenectomy, one by rituximab monotherapy), and one had a prior history of testicular carcinoma treated by orchiectomy and chemotherapy. There was no family history suggestive of inherited bone marrow failure syndromes in any of the cases.

### Treatment and outcomes

Patients presenting with HL and AA sequentially received therapy that generally followed standard practices for each condition. One patient presented with AA before HL and was treated with ATG-based immunosuppression, achieving a complete remission; he subsequently developed HL 13 years after his AA diagnosis and achieved a complete remission with ABVD. Nine patients presented with HL before AA: all patients for whom HL treatment and outcome information was available (five of the five evaluable patients) received standard HL-directed chemotherapy, achieving a complete remission from HL (Table 2). All five patients subsequently died of AA-related complications: two patients received ATG/CsA without response (one subsequently evolved to TRMN, and one died of AA-related complications after salvage allogeneic BMT and graft failure), one patient was treated with CsA and steroids without response and died of hemorrhage, and two patients received supportive care only (Table 2).

In contrast, there was no consensus approach for therapy among the six patients who presented with AA and HL concurrently. Of the five patients with available treatment and outcome information, only one patient achieved a complete remission at 16 months of follow-up: he was treated with immunosuppression with anti-lymphocyte globulin and steroids with persistent cytopenias, followed by MUD-BMT with Cy and total lymphoid irradiation conditioning; his HL was reported to have been in radiographic remission as assessed by thoracic chest computed tomography at the time of transplant. One patient was treated with brentuximab vedotin for HL with progression after eight cycles and with eltrombopag monotherapy for AA without response; he died of neutropenic sepsis after one cycle of salvage ICE chemotherapy. One patient was treated with MOPP (mechlorethamine, vincristine, procarbazine, prednisone) therapy for HL with a complete response of HL and a transient improvement of bone marrow aplasia, followed by a recurrence of AA four months after the patient achieved a complete remission from HL; no additional clinical follow-up is presented in the publication. Two patients were treated with supportive care only.

Overall, of the ten patients who developed AA concurrent with or after HL with available outcome information, eight patients (80%) died of AA-related complications at a median of eight months (range: 1 to 84 months) from the time of AA diagnosis, one patient continued to have AA at the time of the report, and only one patient achieved a complete remission from both AA and HL after Allo BMT (Fig 2, Table 2).

## Discussion

Our data demonstrate that patients with HL have at least a 20-fold higher incidence of AA compared to the general population. We show that in HL patients, AA is significantly more likely to arise concurrently with or after a diagnosis of HL, and thus is probably not linked to previous exposure to cytotoxic chemotherapy. Our results indicate that AA occurs without marrow involvement by HL and in 25% of patients, AA emerges multiple years after achieving a complete remission from HL. We show that outcomes of HL patients who develop AA are extremely poor with current management strategies for AA, with 80% of patients in our study for whom outcome information was available dying from AA-related complications. Taken together, our findings suggest that the occurrence of HL increases the risk of developing AA and underscore the need for improved recognition and treatment of this patient population.

Prospective epidemiologic studies of rare disorders can be logistically prohibitive, particularly for studies of co-occurrence of two very rare diseases such as HL and AA. We acknowledge that it is not possible to estimate with precision the incidence of AA in HL patients based on a retrospective analysis, which can be affected by referral and ascertainment biases. Importantly, despite this limitation, our results provide multiple lines of evidence for a significantly increased incidence of AA in HL patients. First, our results likely underestimate the true incidence of AA in our institutional cohort because not all HL patients continued to receive follow-up at our institution for the full duration of the study period. Second, the prevalence of AA in our institution’s cohort is in agreement with several prospective studies of HL outcomes, where single cases of AA were noted in cohorts of only 130–200 patients [24, 25]. It is likely that some prospective HL studies did not include AA or non-malignant hematologic conditions as a reportable outcome; additionally, clinical trials of HL may not have included HL patients with concurrent AA. Finally, the link between the occurrence of HL and risk of AA is further supported by the striking temporal association between the HL and AA diagnoses, where HL precedes or occurs concurrently with AA in nearly all cases. Only one patient in our study developed AA prior to HL, and to the best of our knowledge there have been no additional reports of HL in patients previously treated for AA neither in individual case reports nor in clinical trials of AA patients. Because of the different order of ocurrence, we speculate that the relationship between AA and HL emergence in Patient 4 is distinct from other cases. Importantly, even after excluding that case, the estimated incidence of AA in HL patients in our study (113 per million per year with 95% CI of [23 per million, 331 per million]) is still significantly higher than the population-based incidence of 2.2 cases per million per year in Western countries to ~7 cases per million per year in Asia (p<0.05) [6]^,^[19].

The mechanism through which HL can predispose to the development of AA is not known. Because immune dysregulation is a feature of both AA and HL, we hypothesize that the link may be due to a HL-related break in immune tolerance, e.g., through cross-reacting immune responses directed against HL or by altering immune regulatory pathways. Importantly, our results argue against several alternative explanations. AA is unlikely to represent a late toxicity related to HL therapy because in 37.5% of HL patients who also developed AA, AA was found concurrently with HL without prior HL-directed treatment. Similarly, AA is unlikely to be a paraneoplastic manifestation of HL because ~25% of patients developed AA after two years or more of complete remission from HL. The strict temporal relationship with HL preceding AA argues against a common infectious trigger or an occult inherited bone marrow failure syndrome, situations in which a predilection for one diagnosis occurring temporally before the other would not be expected. Notably, none of the patients in our cohort received checkpoint inhibitor immunotherapy for HL, the introduction of which could theoretically further increase the incidence of HL-associated AA.

Importantly, our analysis indicates that in contrast to AA in the general population, where five-year overall survival is >80% in pediatric and >60% in adult patients [27], the outcomes of AA in patients with HL diagnosed concurrently or those with a prior history of HL are markedly inferior. Eighty percent of patients in our study for whom survival information is available died from AA-related complications at a median survival of eight months from the time of AA diagnosis. We cannot make definitive conclusions as to the reasons for such poor outcomes, because this would require larger, prospective studies. However, our study raises several potential possibilities. Because AA is rare and remains a diagnosis of exclusion, patients with a history of HL or concurrent HL may have a delay in diagnosis of AA and thus a delay in starting AA-directed therapy; 40% of evaluable patients in our study received supportive care only. Our cohort includes three patients who presented with AA at least a decade after achieving a complete remission with standard cytotoxic therapy for HL and were treated with immunosuppression for AA (two with ATG/CsA and one with CsA/steroids). All three patients were refractory to immunosuppression and died of AA-related complications, including one patient who evolved to TRMN after immunosuppression. We speculate that patients with a prior history of intensive chemotherapy could have a compromised stem cell pool and may have impaired hematopoietic recovery and be predisposed to clonal complications. In fact, both patients from our institution experienced clonal evolution after immunosuppression—one patient developed a new cytogenetic abnormality while the other transformed to a myeloid neoplasm. An alternative explanation for poor response to immunosuppression may be that the etiology of AA, at least in some HL patients, may instead be due to an overlap between AA and hypoplastic MDS [28] or may not be immune-mediated. Finally, the treatment of patients presenting with AA and HL concurrently is particularly challenging. Our results indicate that the majority of this population did not receive standard therapy for either condition and that outcomes for this group are mostly driven by AA-related complications. Novel approaches, perhaps incorporating upfront allogeneic BMT, are needed to improve overall survival in this population.

In conclusion, we have shown that patients with HL have a significantly higher incidence of AA compared to the general population. Our results underscore that HL-associated AA carries a poor prognosis with conventional approaches to therapy. Recognition of this association, both when presenting as a new diagnosis of AA in a patient with a remote history of HL or as a new diagnosis of HL concurrent with severe pancytopenia and bone marrow aplasia, is essential for the institution of optimal therapy and the improvement of outcomes in this population. Future studies are needed to determine the pathogenic mechanisms underlying the development of AA in HL patients, as well as to develop more effective treatment approaches for this rare and difficult-to-treat population.

## Supporting information

S1 Appendix Supporting appendix containing Tables A- C, and Figure A. (DOCX)
