# Supplementary material for: Hodgkin lymphoma patients have an increased incidence of idiopathic acquired aplastic anemia
Source: PLoS One. 2019 Apr 5;14(4):e0215021. doi: 10.1371/journal.pone.0215021 (PMC6450628; doi:10.1371/journal.pone.0215021)

**S1 Appendix**

**Supporting information**

**Title:** Hodgkin lymphoma patients have an increased incidence of idiopathic acquired aplastic anemia

**Short Running Title:** Aplastic anemia in Hodgkin lymphoma patients

Taylor Linaburg^1^, Adam R. Davis^2^ Noelle V. Frey^3^, Muhammad R. Khawaja^4^, Daniel J. Landsburg^3^, Stephen J. Schuster^3^, Jakub Svoboda^3^, Yimei Li^5^, Yuliya Borovskiy^6^, Timothy S. Olson^7^, Adam Bagg^2^, Elizabeth O. Hexner^3^, Daria V. Babushok^3*^

^1^ Perelman School of Medicine, University of Pennsylvania, Philadelphia, PA;

^2^ Department of Pathology and Laboratory Medicine, Hospital of the University of Pennsylvania, Philadelphia, Philadelphia, PA;

^3^ Division of Hematology-Oncology, Department of Medicine, Hospital of the University of Pennsylvania, Philadelphia, PA;

^4^ Division of Hematology-Oncology, Milton S. Hershey Medical Center, Penn State Cancer Institute, Hershey, PA

^5^ Department of Biostatistics and Epidemiology, Perelman School of Medicine at the University of Pennsylvania, Philadelphia, PA;

^6^ Penn Medicine Corporate Information Services, University of Pennsylvania Health System, Philadelphia, PA;

^7^ Division of Oncology, Department of Pediatrics, Children’s Hospital of Philadelphia, Philadelphia, PA

* Corresponding Author:

Email: daria.babushok@uphs.upenn.edu (DB)

**Table of Contents**

[**Supporting Tables** 3](#_Toc536358611)

[**Table A: ICD9 and ICD10 codes used for electronic medical record search of aplastic anemia and pancytopenia** 3](#_Toc536358612)

[**Table B: ICD9 and ICD10 codes used for electronic medical record search of chronic lymphocytic lymphoma** 4](#_Toc536358613)

[**Table C: ICD9 and ICD10 codes used for electronic medical record search of Hodgkin lymphoma** 5](#_Toc536358614)

[**Supporting Figures** 11](#_Toc536358615)

[**Figure A. A systematic review and quality analysis of patients with AA with a prior diagnosis of Hodgkin lymphoma** 11](#_Toc536358616)

# **Supporting Tables**

## **Table A: ICD9 and ICD10 codes used for electronic medical record search of aplastic anemia and pancytopenia**

| Code | Category | ICD | Long Description |
| --- | --- | --- | --- |
| 284.09 | Pancytopenia | ICD9 | Other constitutional aplastic anemia |
| 284.19 | Pancytopenia | ICD9 | Other pancytopenia |
| 284.89 | Pancytopenia | ICD9 | Other specified aplastic anemias |
| 284.9 | Pancytopenia | ICD9 | Aplastic anemia, unspecified |
| D61.09 | Pancytopenia | ICD10 | Other constitutional aplastic anemia |
| D61.2 | Pancytopenia | ICD10 | Aplastic anemia due to other external agents |
| D61.3 | Pancytopenia | ICD10 | Idiopathic aplastic anemia |
| D61.818 | Pancytopenia | ICD10 | Other pancytopenia |
| D61.89 | Pancytopenia | ICD10 | Other specified aplastic anemias and other bone marrow failure syndromes |
| D61.9 | Pancytopenia | ICD10 | Aplastic anemia, unspecified |

## **Table B: ICD9 and ICD10 codes used for electronic medical record search of chronic lymphocytic lymphoma**

| Code | Category | ICD | Long Description |
| --- | --- | --- | --- |
| C91.10 | CLL | ICD10 | Chronic lymphocytic leukemia of B-cell type not having achieved remission |
| C91.11 | CLL | ICD10 | Chronic lymphocytic leukemia of B-cell type in remission |
| C91.12 | CLL | ICD10 | Chronic lymphocytic leukemia of B-cell type in relapse |
| C83.00 | CLL | ICD10 | Small cell B-cell lymphoma, unspecified site |
| C83.01 | CLL | ICD10 | Small cell B-cell lymphoma, lymph nodes of head, face, and neck |
| C83.02 | CLL | ICD10 | Small cell B-cell lymphoma, intrathoracic lymph nodes |
| C83.03 | CLL | ICD10 | Small cell B-cell lymphoma, intra-abdominal lymph nodes |
| C83.04 | CLL | ICD10 | Small cell B-cell lymphoma, lymph nodes of axilla and upper limb |
| C83.05 | CLL | ICD10 | Small cell B-cell lymphoma, lymph nodes of inguinal region and lower limb |
| C83.06 | CLL | ICD10 | Small cell B-cell lymphoma, intrapelvic lymph nodes |
| C83.07 | CLL | ICD10 | Small cell B-cell lymphoma, spleen |
| C83.08 | CLL | ICD10 | Small cell B-cell lymphoma, lymph nodes of multiple sites |
| C83.09 | CLL | ICD10 | Small cell B-cell lymphoma, extranodal and solid organ sites |

## **Table C: ICD9 and ICD10 codes used for electronic medical record search of Hodgkin lymphoma**

| Code | Category | ICD | Long Description |
| --- | --- | --- | --- |
| C81.00 | Hodgkin | ICD10 | Nodular lymphocyte predominant Hodgkin lymphoma, unspecified site |
| C81.01 | Hodgkin | ICD10 | Nodular lymphocyte predominant Hodgkin lymphoma, lymph nodes of head, face, and neck |
| C81.02 | Hodgkin | ICD10 | Nodular lymphocyte predominant Hodgkin lymphoma, intrathoracic lymph nodes |
| C81.03 | Hodgkin | ICD10 | Nodular lymphocyte predominant Hodgkin lymphoma, intra-abdominal lymph nodes |
| C81.04 | Hodgkin | ICD10 | Nodular lymphocyte predominant Hodgkin lymphoma, lymph nodes of axilla and upper limb |
| C81.05 | Hodgkin | ICD10 | Nodular lymphocyte predominant Hodgkin lymphoma, lymph nodes of inguinal region and lower limb |
| C81.06 | Hodgkin | ICD10 | Nodular lymphocyte predominant Hodgkin lymphoma, intrapelvic lymph nodes |
| C81.07 | Hodgkin | ICD10 | Nodular lymphocyte predominant Hodgkin lymphoma, spleen |
| C81.08 | Hodgkin | ICD10 | Nodular lymphocyte predominant Hodgkin lymphoma, lymph nodes of multiple sites |
| C81.09 | Hodgkin | ICD10 | Nodular lymphocyte predominant Hodgkin lymphoma, extranodal and solid organ sites |
| C81.10 | Hodgkin | ICD10 | Nodular sclerosis Hodgkin lymphoma, unspecified site |
| C81.11 | Hodgkin | ICD10 | Nodular sclerosis Hodgkin lymphoma, lymph nodes of head, face, and neck |
| C81.12 | Hodgkin | ICD10 | Nodular sclerosis Hodgkin lymphoma, intrathoracic lymph nodes |
| C81.13 | Hodgkin | ICD10 | Nodular sclerosis Hodgkin lymphoma, intra-abdominal lymph nodes |
| C81.14 | Hodgkin | ICD10 | Nodular sclerosis Hodgkin lymphoma, lymph nodes of axilla and upper limb |
| C81.15 | Hodgkin | ICD10 | Nodular sclerosis Hodgkin lymphoma, lymph nodes of inguinal region and lower limb |
| C81.16 | Hodgkin | ICD10 | Nodular sclerosis Hodgkin lymphoma, intrapelvic lymph nodes |
| C81.17 | Hodgkin | ICD10 | Nodular sclerosis Hodgkin lymphoma, spleen |
| C81.18 | Hodgkin | ICD10 | Nodular sclerosis Hodgkin lymphoma, lymph nodes of multiple sites |
| C81.19 | Hodgkin | ICD10 | Nodular sclerosis Hodgkin lymphoma, extranodal and solid organ sites |
| C81.20 | Hodgkin | ICD10 | Mixed cellularity Hodgkin lymphoma, unspecified site |
| C81.21 | Hodgkin | ICD10 | Mixed cellularity Hodgkin lymphoma, lymph nodes of head, face, and neck |
| C81.22 | Hodgkin | ICD10 | Mixed cellularity Hodgkin lymphoma, intrathoracic lymph nodes |
| C81.23 | Hodgkin | ICD10 | Mixed cellularity Hodgkin lymphoma, intra-abdominal lymph nodes |
| C81.24 | Hodgkin | ICD10 | Mixed cellularity Hodgkin lymphoma, lymph nodes of axilla and upper limb |
| C81.25 | Hodgkin | ICD10 | Mixed cellularity Hodgkin lymphoma, lymph nodes of inguinal region and lower limb |
| C81.26 | Hodgkin | ICD10 | Mixed cellularity Hodgkin lymphoma, intrapelvic lymph nodes |
| C81.27 | Hodgkin | ICD10 | Mixed cellularity Hodgkin lymphoma, spleen |
| C81.28 | Hodgkin | ICD10 | Mixed cellularity Hodgkin lymphoma, lymph nodes of multiple sites |
| C81.29 | Hodgkin | ICD10 | Mixed cellularity Hodgkin lymphoma, extranodal and solid organ sites |
| C81.30 | Hodgkin | ICD10 | Lymphocyte depleted Hodgkin lymphoma, unspecified site |
| C81.31 | Hodgkin | ICD10 | Lymphocyte depleted Hodgkin lymphoma, lymph nodes of head, face, and neck |
| C81.32 | Hodgkin | ICD10 | Lymphocyte depleted Hodgkin lymphoma, intrathoracic lymph nodes |
| C81.33 | Hodgkin | ICD10 | Lymphocyte depleted Hodgkin lymphoma, intra-abdominal lymph nodes |
| C81.34 | Hodgkin | ICD10 | Lymphocyte depleted Hodgkin lymphoma, lymph nodes of axilla and upper limb |
| C81.35 | Hodgkin | ICD10 | Lymphocyte depleted Hodgkin lymphoma, lymph nodes of inguinal region and lower limb |
| C81.36 | Hodgkin | ICD10 | Lymphocyte depleted Hodgkin lymphoma, intrapelvic lymph nodes |
| C81.37 | Hodgkin | ICD10 | Lymphocyte depleted Hodgkin lymphoma, spleen |
| C81.38 | Hodgkin | ICD10 | Lymphocyte depleted Hodgkin lymphoma, lymph nodes of multiple sites |
| C81.39 | Hodgkin | ICD10 | Lymphocyte depleted Hodgkin lymphoma, extranodal and solid organ sites |
| C81.40 | Hodgkin | ICD10 | Lymphocyte-rich Hodgkin lymphoma, unspecified site |
| C81.41 | Hodgkin | ICD10 | Lymphocyte-rich Hodgkin lymphoma, lymph nodes of head, face, and neck |
| C81.42 | Hodgkin | ICD10 | Lymphocyte-rich Hodgkin lymphoma, intrathoracic lymph nodes |
| C81.43 | Hodgkin | ICD10 | Lymphocyte-rich Hodgkin lymphoma, intra-abdominal lymph nodes |
| C81.44 | Hodgkin | ICD10 | Lymphocyte-rich Hodgkin lymphoma, lymph nodes of axilla and upper limb |
| C81.45 | Hodgkin | ICD10 | Lymphocyte-rich Hodgkin lymphoma, lymph nodes of inguinal region and lower limb |
| C81.46 | Hodgkin | ICD10 | Lymphocyte-rich Hodgkin lymphoma, intrapelvic lymph nodes |
| C81.47 | Hodgkin | ICD10 | Lymphocyte-rich Hodgkin lymphoma, spleen |
| C81.48 | Hodgkin | ICD10 | Lymphocyte-rich Hodgkin lymphoma, lymph nodes of multiple sites |
| C81.49 | Hodgkin | ICD10 | Lymphocyte-rich Hodgkin lymphoma, extranodal and solid organ sites |
| C81.70 | Hodgkin | ICD10 | Other Hodgkin lymphoma, unspecified site |
| C81.71 | Hodgkin | ICD10 | Other Hodgkin lymphoma, lymph nodes of head, face, and neck |
| C81.72 | Hodgkin | ICD10 | Other Hodgkin lymphoma, intrathoracic lymph nodes |
| C81.73 | Hodgkin | ICD10 | Other Hodgkin lymphoma, intra-abdominal lymph nodes |
| C81.74 | Hodgkin | ICD10 | Other Hodgkin lymphoma, lymph nodes of axilla and upper limb |
| C81.75 | Hodgkin | ICD10 | Other Hodgkin lymphoma, lymph nodes of inguinal region and lower limb |
| C81.76 | Hodgkin | ICD10 | Other Hodgkin lymphoma, intrapelvic lymph nodes |
| C81.77 | Hodgkin | ICD10 | Other Hodgkin lymphoma, spleen |
| C81.78 | Hodgkin | ICD10 | Other Hodgkin lymphoma, lymph nodes of multiple sites |
| C81.79 | Hodgkin | ICD10 | Other Hodgkin lymphoma, extranodal and solid organ sites |
| C81.90 | Hodgkin | ICD10 | Hodgkin lymphoma, unspecified, unspecified site |
| C81.91 | Hodgkin | ICD10 | Hodgkin lymphoma, unspecified, lymph nodes of head, face, and neck |
| C81.92 | Hodgkin | ICD10 | Hodgkin lymphoma, unspecified, intrathoracic lymph nodes |
| C81.93 | Hodgkin | ICD10 | Hodgkin lymphoma, unspecified, intra-abdominal lymph nodes |
| C81.94 | Hodgkin | ICD10 | Hodgkin lymphoma, unspecified, lymph nodes of axilla and upper limb |
| C81.95 | Hodgkin | ICD10 | Hodgkin lymphoma, unspecified, lymph nodes of inguinal region and lower limb |
| C81.96 | Hodgkin | ICD10 | Hodgkin lymphoma, unspecified, intrapelvic lymph nodes |
| C81.97 | Hodgkin | ICD10 | Hodgkin lymphoma, unspecified, spleen |
| C81.98 | Hodgkin | ICD10 | Hodgkin lymphoma, unspecified, lymph nodes of multiple sites |
| C81.99 | Hodgkin | ICD10 | Hodgkin lymphoma, unspecified, extranodal and solid organ sites |
| 201 | Hodgkin | ICD9 | Hodgkin's paragranuloma, unspecified site, extranodal and solid organ sites |
| 201.01 | Hodgkin | ICD9 | Hodgkin's paragranuloma, lymph nodes of head, face, and neck |
| 201.02 | Hodgkin | ICD9 | Hodgkin's paragranuloma, intrathoracic lymph nodes |
| 201.03 | Hodgkin | ICD9 | Hodgkin's paragranuloma, intra-abdominal lymph nodes |
| 201.04 | Hodgkin | ICD9 | Hodgkin's paragranuloma, lymph nodes of axilla and upper limb |
| 201.05 | Hodgkin | ICD9 | Hodgkin's paragranuloma, lymph nodes of inguinal region and lower limb |
| 201.06 | Hodgkin | ICD9 | Hodgkin's paragranuloma, intrapelvic lymph nodes |
| 201.07 | Hodgkin | ICD9 | Hodgkin's paragranuloma, spleen |
| 201.08 | Hodgkin | ICD9 | Hodgkin's paragranuloma, lymph nodes of multiple sites |
| 201.1 | Hodgkin | ICD9 | Hodgkin's granuloma, unspecified site, extranodal and solid organ sites |
| 201.11 | Hodgkin | ICD9 | Hodgkin's granuloma, lymph nodes of head, face, and neck |
| 201.12 | Hodgkin | ICD9 | Hodgkin's granuloma, intrathoracic lymph nodes |
| 201.13 | Hodgkin | ICD9 | Hodgkin's granuloma, intra-abdominal lymph nodes |
| 201.14 | Hodgkin | ICD9 | Hodgkin's granuloma, lymph nodes of axilla and upper limb |
| 201.15 | Hodgkin | ICD9 | Hodgkin's granuloma, lymph nodes of inguinal region and lower limb |
| 201.16 | Hodgkin | ICD9 | Hodgkin's granuloma, intrapelvic lymph nodes |
| 201.17 | Hodgkin | ICD9 | Hodgkin's granuloma, spleen |
| 201.18 | Hodgkin | ICD9 | Hodgkin's granuloma, lymph nodes of multiple sites |
| 201.2 | Hodgkin | ICD9 | Hodgkin's sarcoma, unspecified site, extranodal and solid organ sites |
| 201.21 | Hodgkin | ICD9 | Hodgkin's sarcoma, lymph nodes of head, face, and neck |
| 201.22 | Hodgkin | ICD9 | Hodgkin's sarcoma, intrathoracic lymph nodes |
| 201.23 | Hodgkin | ICD9 | Hodgkin's sarcoma, intra-abdominal lymph nodes |
| 201.24 | Hodgkin | ICD9 | Hodgkin's sarcoma, lymph nodes of axilla and upper limb |
| 201.25 | Hodgkin | ICD9 | Hodgkin's sarcoma, lymph nodes of inguinal region and lower limb |
| 201.26 | Hodgkin | ICD9 | Hodgkin's sarcoma, intrapelvic lymph nodes |
| 201.27 | Hodgkin | ICD9 | Hodgkin's sarcoma, spleen |
| 201.28 | Hodgkin | ICD9 | Hodgkin's sarcoma, lymph nodes of multiple sites |
| 201.4 | Hodgkin | ICD9 | Hodgkin's disease, lymphocytic-histiocytic predominance, unspecified site, extranodal and solid organ sites |
| 201.41 | Hodgkin | ICD9 | Hodgkin's disease, lymphocytic-histiocytic predominance, lymph nodes of head, face, and neck |
| 201.42 | Hodgkin | ICD9 | Hodgkin's disease, lymphocytic-histiocytic predominance, intrathoracic lymph nodes |
| 201.43 | Hodgkin | ICD9 | Hodgkin's disease, lymphocytic-histiocytic predominance, intra-abdominal lymph nodes |
| 201.44 | Hodgkin | ICD9 | Hodgkin's disease, lymphocytic-histiocytic predominance, lymph nodes of axilla and upper limb |
| 201.45 | Hodgkin | ICD9 | Hodgkin's disease, lymphocytic-histiocytic predominance, lymph nodes of inguinal region and lower limb |
| 201.46 | Hodgkin | ICD9 | Hodgkin's disease, lymphocytic-histiocytic predominance, intrapelvic lymph nodes |
| 201.47 | Hodgkin | ICD9 | Hodgkin's disease, lymphocytic-histiocytic predominance, spleen |
| 201.48 | Hodgkin | ICD9 | Hodgkin's disease, lymphocytic-histiocytic predominance, lymph nodes of multiple sites |
| 201.5 | Hodgkin | ICD9 | Hodgkin's disease, nodular sclerosis, unspecified site, extranodal and solid organ sites |
| 201.51 | Hodgkin | ICD9 | Hodgkin's disease, nodular sclerosis, lymph nodes of head, face, and neck |
| 201.52 | Hodgkin | ICD9 | Hodgkin's disease, nodular sclerosis, intrathoracic lymph nodes |
| 201.53 | Hodgkin | ICD9 | Hodgkin's disease, nodular sclerosis, intra-abdominal lymph nodes |
| 201.54 | Hodgkin | ICD9 | Hodgkin's disease, nodular sclerosis, lymph nodes of axilla and upper limb |
| 201.55 | Hodgkin | ICD9 | Hodgkin's disease, nodular sclerosis, lymph nodes of inguinal region and lower limb |
| 201.56 | Hodgkin | ICD9 | Hodgkin's disease, nodular sclerosis, intrapelvic lymph nodes |
| 201.57 | Hodgkin | ICD9 | Hodgkin's disease, nodular sclerosis, spleen |
| 201.58 | Hodgkin | ICD9 | Hodgkin's disease, nodular sclerosis, lymph nodes of multiple sites |
| 201.6 | Hodgkin | ICD9 | Hodgkin's disease, mixed cellularity, unspecified site, extranodal and solid organ sites |
| 201.61 | Hodgkin | ICD9 | Hodgkin's disease, mixed cellularity, lymph nodes of head, face, and neck |
| 201.62 | Hodgkin | ICD9 | Hodgkin's disease, mixed cellularity, intrathoracic lymph nodes |
| 201.63 | Hodgkin | ICD9 | Hodgkin's disease, mixed cellularity, intra-abdominal lymph nodes |
| 201.64 | Hodgkin | ICD9 | Hodgkin's disease, mixed cellularity, lymph nodes of axilla and upper limb |
| 201.65 | Hodgkin | ICD9 | Hodgkin's disease, mixed cellularity, lymph nodes of inguinal region and lower limb |
| 201.66 | Hodgkin | ICD9 | Hodgkin's disease, mixed cellularity, intrapelvic lymph nodes |
| 201.67 | Hodgkin | ICD9 | Hodgkin's disease, mixed cellularity, spleen |
| 201.68 | Hodgkin | ICD9 | Hodgkin's disease, mixed cellularity, lymph nodes of multiple sites |
| 201.7 | Hodgkin | ICD9 | Hodgkin's disease, lymphocytic depletion, unspecified site, extranodal and solid organ sites |
| 201.71 | Hodgkin | ICD9 | Hodgkin's disease, lymphocytic depletion, lymph nodes of head, face, and neck |
| 201.72 | Hodgkin | ICD9 | Hodgkin's disease, lymphocytic depletion, intrathoracic lymph nodes |

# **Supporting Figures**

## **Figure A. A systematic review and quality analysis of patients with AA with a prior diagnosis of Hodgkin lymphoma**


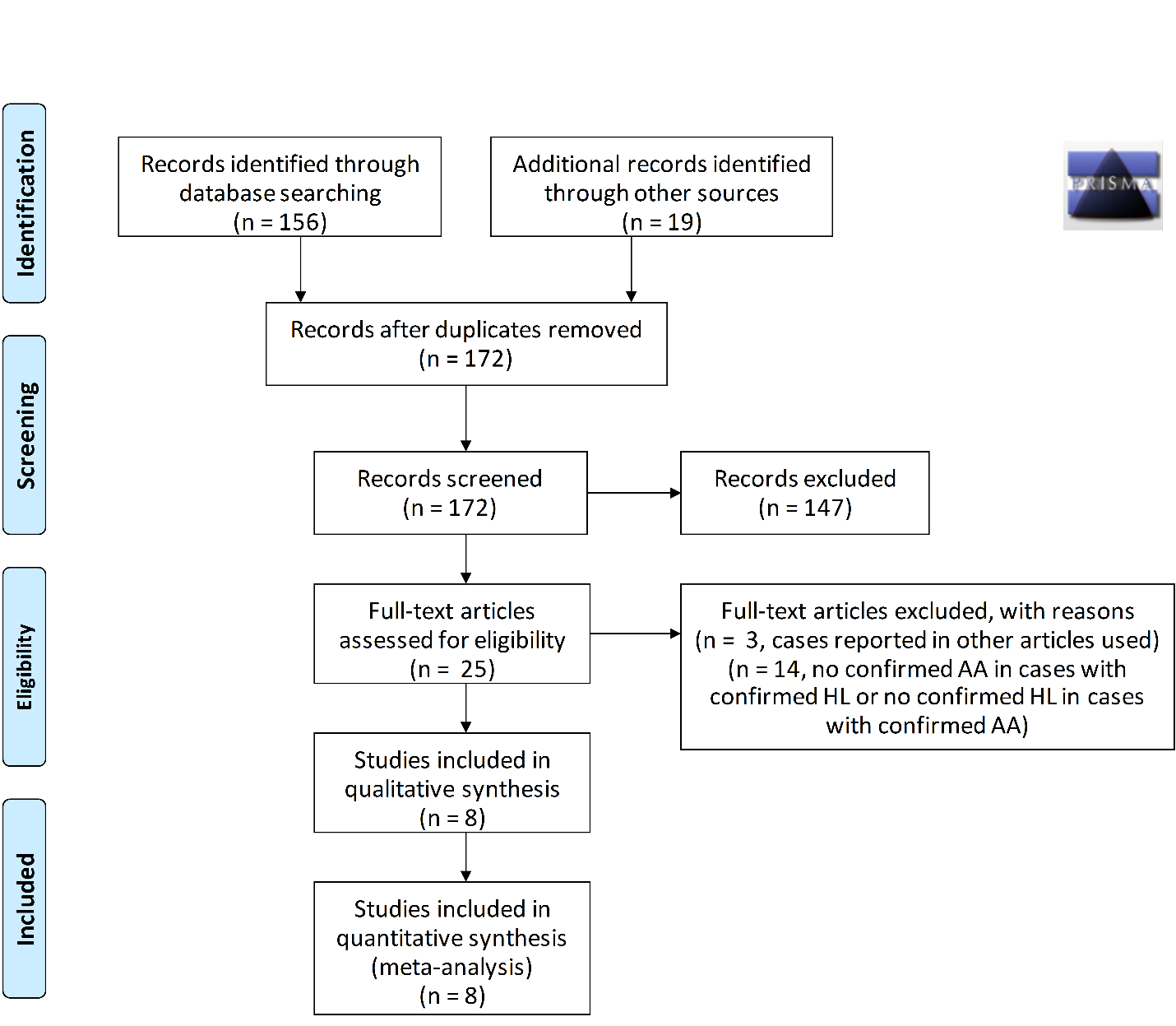

Supplement: S1 Appendix — (DOCX) [file pone.0215021.s001.docx]
